# Pathophysiology, diagnosis and treatment of tachycardiomyopathy

**DOI:** 10.1136/heartjnl-2016-310391

**Published:** 2017-08-30

**Authors:** Claire A Martin, Pier D Lambiase

**Affiliations:** Department of Cardiology, Barts Health NHS Trust, London, UK

**Keywords:** atrial arrhythmia ablation procedures, ventricular arrhythmia ablation procedures, cardiac magnetic resonance (cmr) imaging, idiopathic dilated cardiomyopathy, heart failure

**Learning objectives**

- Recognise the diagnosis of tachycardiomyopathy (TCMP)
- Understand the pathophysiology
- Determine treatment strategies to restore left ventricular function
- The role of TCMP in non-responders to cardiac resynchronisation

## Introduction

**Figure 1:**
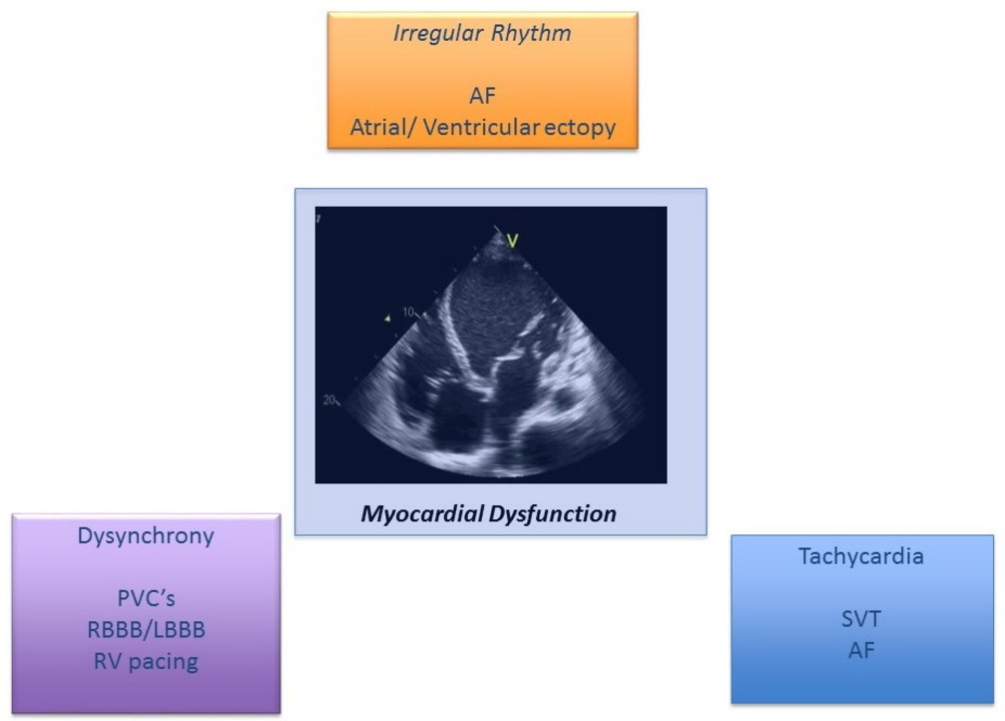
Arrhythmias leading to left ventricular dysfunction. AF, atrial fibrillation; LBBB, left bundle branch block; PVCs, premature ventricular complexes; RBBB, right bundle branch block; RV, right ventricular; SVT, supraventricular tachycardia.

Tachycardiomyopathies (TCMP) are an important cause of left ventricular (LV) dysfunction that should be recognised by physicians as they are potentially reversible and have a significant impact on morbidity and prognosis. They are classically defined as the reversible impairment of ventricular function induced by persistent arrhythmia. However, it is becoming increasingly evident that they can be induced by atrial and ventricular ectopy promoting dyssynchrony and indeed the term ‘arrhythmia-induced cardiomyopathy’ is emerging to describe the phenomenon.^1 2^ A more current proposed definition highlights aetiology: *‘Atrial and/or ventricular dysfunction—secondary to rapid and/or asynchronous/irregular myocardial contraction, partially or completely reversed after treatment of the causative arrhythmia’* 3 (figure 1). Two categories of the condition exist: the arrhythmia is the only reason for ventricular dysfunction (*arrhythmia-induced*), and another where the arrhythmia exacerbates ventricular dysfunction and/or worsens heart failure (HF) in a patient with concomitant heart disease (*arrhythmia-mediated*).^4^ The exclusion of underlying structural heart disease can be challenging as current imaging techniques, for example, MRI cannot easily identify diffuse fibrosis which may itself be primary or secondary to the effects of arrhythmia promoting ventricular wall dyskinesis and stretch or valvular regurgitation.

## Pathophysiology

**Figure 2:**
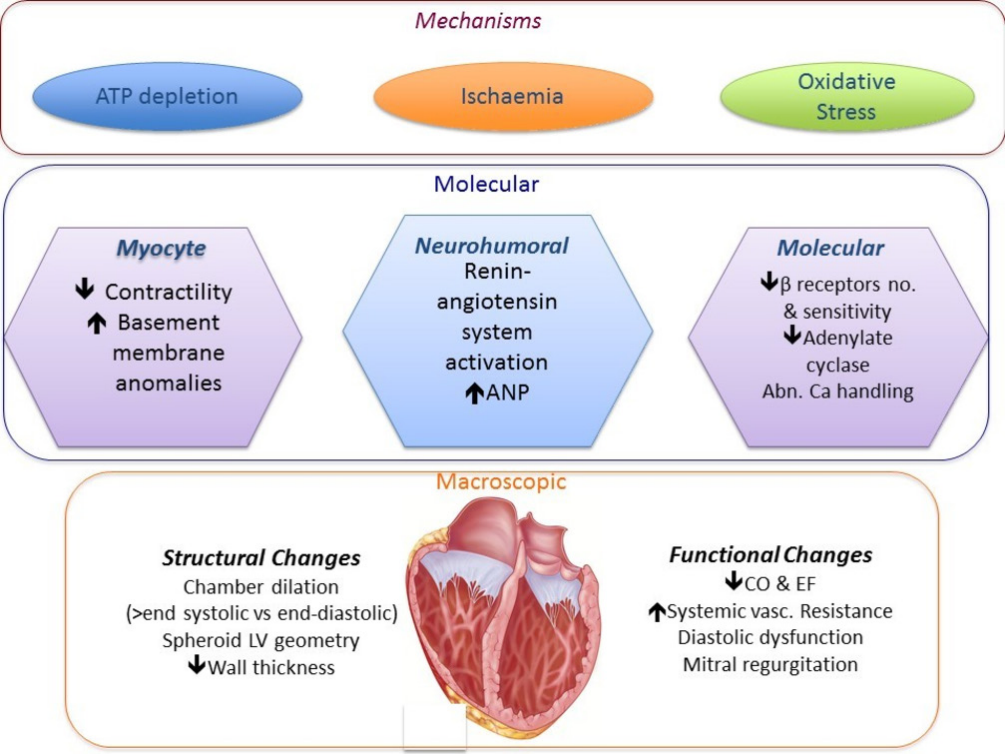
Mechanisms of tachycardiomyopathy (TCMP). The molecular, microscopic and structural effects of TCMP.

The mechanisms of TCMP are not fully defined but include subclinical ischaemia, abnormalities in energy metabolism, redox stress and calcium overload.^5 6^ In animal models of persistent high rate atrial or ventricular pacing, ventricular impairment is also associated with changes in myocardial electrophysiology including prolongation of the action potential and spontaneous ventricular arrhythmias. Indeed, persistent left bundle branch block leads to lateralisation of gap junctions promoting functional anisotropy and apoptosis.^7^ This can be reversed by LV pacing in HF models. These molecular and cellular changes lead to abnormalities in chamber geometry and negative ventricular modelling (figure 2). It is this reversibility of ventricular function in these disorders that can be remedied by treating the *primary tachycardia,* which makes TCMP important to identify and treat promptly.

## Management

A timely diagnosis of TCMP is important given the potential for recovery with appropriate treatment. The possibility of TCMP should be considered when eliciting a history of any new diagnosis of LV dysfunction, if there is evidence of persistent or frequently occurring tachycardia or frequent premature ventricular complexes (PVCs).^8^ The classic clinical presentation is with symptoms and signs of congestive HF and dilated cardiomyopathy.^9^ It should be noted that patients may not necessarily present with an arrhythmia, therefore a high index of suspicion needs to be maintained. Patients may be diagnosed through echocardiography prior to the onset of clinical symptoms or after developing progressive HF.

It has been suggested that chronic tachycardia that occurs >10%–15% of the day may result in cardiomyopathy.^2^ There is no precise ventricular rate known to lead to TCMP, although rates above 100 bpm are generally thought to be deleterious.^5^

However, as explained above, in the case of atrial fibrillation (AF) or PVCs, it is not only a high heart rate (HR), but also asynchronous myocardial contraction, that can lead to LV dysfunction.The resting HR may not give an indication of the mean HR, as there may be an exaggerated response to exertion, so ambulatory monitoring is important for diagnosis as well as identifying subclinical arrhythmias.

Other factors that point to a diagnosis of TCMP include: (i) evidence of a previously normal ejection fraction (EF) and a degree of LV dysfunction out of proportion to other comorbidities, (ii) no other cause of non-ischaemic cardiomyopathy found (eg, hypertension, alcohol or drug use, stress, etc), (iii) absence of left ventricular hypertrophy, (iv) relatively normal LV dimensions (LV end-diastolic dimension below 5.5 cm), (v) recovery of LV function after control of tachycardia (by rate control, cardioversion or radiofrequency ablation within 1–6 months) and (vi) rapid decline in LV ejection fraction (LVEF) following recurrence of tachycardia in a patient with recovered LV function after previous control of tachycardia.

While the classic definition of TCMP refers to an impairment of LV function in the absence of structural heart disease, in practice patients with pre-existing ventricular dysfunction often deteriorate in the face of an uncontrolled tachycardia, and therefore the role of the tachycardia should be taken into account. It may be difficult in such circumstances to distinguish whether the arrhythmia or the cardiomyopathy is the primary driver. Cardiac imaging provides important information in TCMP to identify underlying structural disease—patients initially have smaller LV end-diastolic diameters and LV volume adjusted for body surface area and LV mass compared with patients with idiopathic dilated cardiomyopathy.^10^ The presence of gadolinium late enhancement can be an indicator of structural pathology pointing to a reduced likelihood to response in the context of PVC ablation.^11^ In an electro-anatomical mapping study of PVC cases, patients with irreversible cardiomyopathy had greater areas of low amplitude signals, a reduced unipolar voltage area ≥32% of LV endocardium predicted the irreversibility of cardiomyopathy with >95% sensitivity and specificity, but prospective validation is lacking.^12^

Serial assessment of the N-terminal pro-B-type natriuretic peptide (NT-proBNP) ratio (NT-BNP at baseline/NT-BNP during follow-up) can differentiate TCMP from irreversible dilated cardiomyopathy. A prompt decline in NT-proBNP levels, after direct currect (DC) cardioversion of AF was associated with reversible cardiomyopathy with an accuracy of 90% in one series.^13^

The diagnosis of TCMP may be evident only after restoration and maintenance of sinus rhythm, or after aggressive rate control meaning a two-pronged approach to treat the arrhythmia and cardiac dysfunction is required.

Once the diagnosis of TCMP has been made, many authors advocate a pro-active treatment approach. The exact strategy employed is dependent on the causative arrhythmia, as detailed below, where either a rate or rhythm control strategy may be more appropriate.^8^ Potentially curative ablation is often the treatment of choice, especially for SVTs, and also AF, ventricular tachycardia (VT) and PVCs.

## Arrhythmias

**Figure 3:**
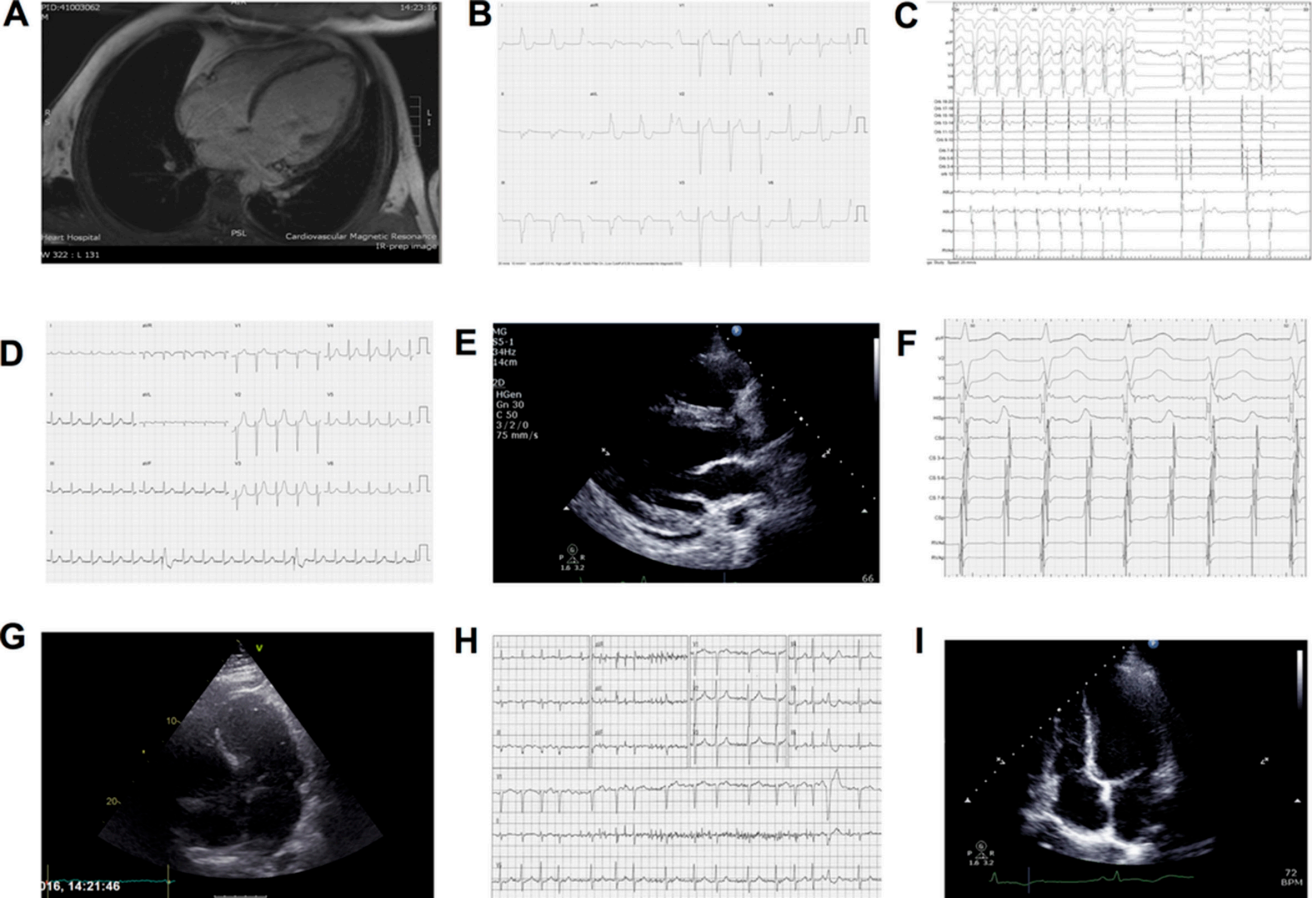
Case vignettes. (1) A man aged 33 years presented complaining of a pulsation in his neck but no other symptoms. (A) MRI demonstrated a markedly dilated left ventricle (LV) and severely impaired biventricular function with extensive mid-wall fibrosis in keeping with a dilated cardiomyopathy. Coronary angiography was normal. (B) ECG demonstrated an incessant idiopathic ventricular rhythm. Electrophysiological studies (EPS) demonstrated a focal source at the right ventricular inflow (note the left bundle branch block pattern with Q waves in the inferior leads indicative of this focus). (C) Ablation at this location terminated the tachycardia and at follow-up his LV function had normalised. (2) A female aged 25 years suffered a cardiac arrest post partum, having had a 2-week preceding history of incessant palpitations. (D) ECG demonstrated a regular narrow complex tachycardia, which was terminated with a synchronised shock. (E) Echocardiogram demonstrated a dilated and severely impaired LV. (F) EPS demonstrated AVNRT with 2:1 and 1:1 conduction. Slow pathway modification terminated the tachycardia and at follow-up LV function has normalised.  (3) A man aged 54 years presented with a 3-month history of rapidly deteriorating breathlessness and palpitations. (G) MRI demonstrated dilated and severely impaired LV (see supplementary file video 1). (H) ECG demonstrated AF with a fast ventricular response. He was rate-controlled with beta-blockers and underwent urgent ablation comprising bilateral wide area circumferential ablation, roof line and mitral isthmus line. He has subsequently maintained sinus rhythm and his LV function has gradually normalised (I) (see online supplementary video 2).

The first report of a man with dilated cardiomyopathy resulting from rapid AF was by Gossage in 1913.^14^ Since then, numerous studies have demonstrated that multiple forms of tachycardia may result in TCMP; these include AF, atrial flutter, incessant SVT, VT and PVCs^15–19^ (figure 1). Restoring sinus rhythm, controlling ventricular response and decreasing the burden of ventricular ectopics can all lead to improvement in LV function and symptoms of HF. TCMP may occur at any age, with cases reported in utero^20^ and in infants and children,^21–23^ as well as in adults (figure 3 illustrates clinical examples). The true incidence is unknown as TCMP is a diagnosis of exclusion, and is therefore likely to be under-recognised in clinical practice—reported series describe incidences of 8%–28% in focal/ectopic atrial tachycardias and 9%–34% for ventricular ectopy and non-sustained VT.^11 18 24–27^ Most reports in the literature involve small retrospective series or individual case reports.

10.1136/heartjnl-2016-310391.supp1 Supplementary file 1 sp1

10.1136/heartjnl-2016-310391.supp2 Supplementary file 2 sp2

## Supraventricular arrhythmias

Whipple *et al* 28 were the first to demonstrate that rapid atrial pacing can induce impairment in ventricular function. Persistent AF is the most studied arrhythmia associated with TCMP, as it is chronic and has a high prevalence. Persistent AF is known to be associated with an increased risk of HF. LV function improves with any of a number of AF treatment strategies, whether that be rate control through medication or a ‘pace and ablate’ procedure (AVN ablation and single chamber VVI pacing), or rhythm control with cardioversion, anti-arrhythmic drugs or catheter ablation.^29–33^ Dissecting whether the LV impairment is due to underlying structural heart disease or the lack of atrial transport and rapid, irregular ventricular rates in their own right is difficult.^32 34^ A ‘pace and ablate’ procedure improves LV function even if the ventricular response was well controlled prior, showing that regularity is likely to be important as well as rate.^35^ However, this approach results in LV dysynchrony and is usually a last resort reserved for elderly patients intolerant of medications.

**Figure 4:**
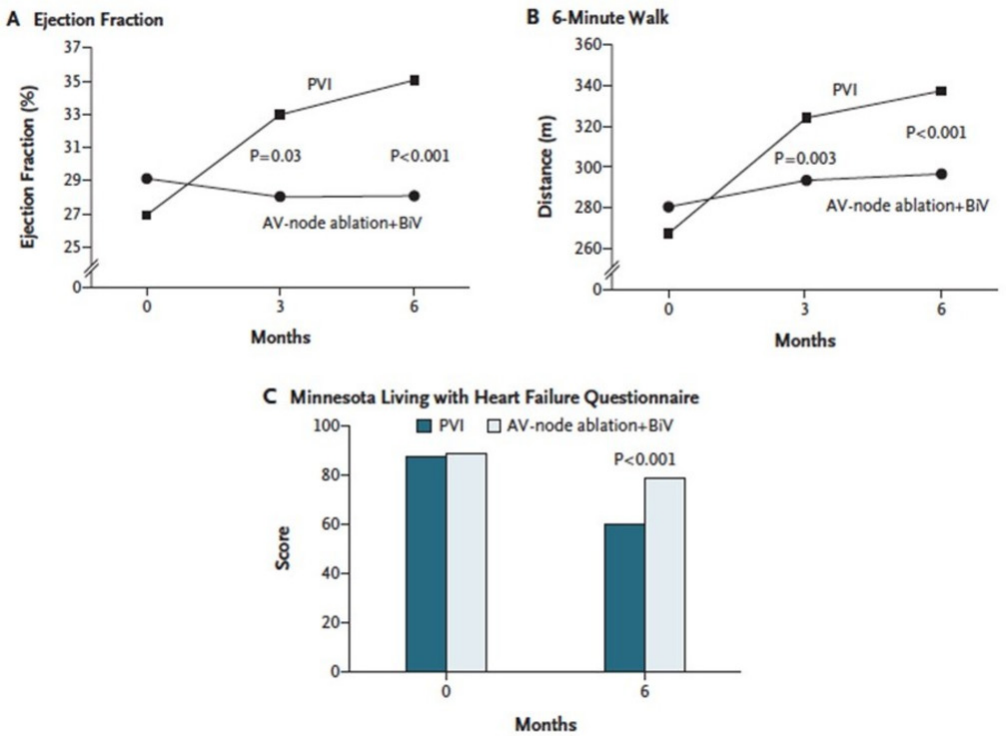
Effect of pulmonary vein isolation (PVI) vs AVN ablation+cardiac resynchronisation on left ventricular function and exercise tolerance in the Pulmonary Vein Antrum Isolation versus AV Node Ablation with Bi-Ventricular Pacing for Treatment of Atrial Fibrillation in Patients with Congestive Heart Failure (PABA CHF) trial. From Khan *et al*.^37^

Randomised studies of AF ablation do demonstrate positive effects on LV remodelling once sinus rhythm is re-established over and above pure rate control. Restoration of sinus rhythm with pulmonary vein isolation was superior to AV node ablation and cardiac resynchronisation (CRT), where the effects of single chamber VVI pacing are minimised (figure 4).^36 37^ In the recent AATAC-AF trial which randomised 203 persistent AF patients with HF and cardiomyopathy (LVEF<40%) to either amiodarone or catheter ablation, 70% of patients in the ablation arm were free of AT/AF (vs 34% in the amiodarone arm (p<0.001)) and had significant improvements in mortality, hospitalisation rates and quality of life. LVEF improved 9.6%±7.4% in the ablation arm versus 4.2%±6.2% in the amiodarone arm (p<0.01).^38^ This is supported by a systematic review of 19 studies (914 patients) evaluating AF ablation in patients with concomitant LV dysfunction where LVEF increased by 13.3% (95% CI 11% to 16%), with 57% maintenance sinus rhythm, after a single procedure and 82% after >1 procedure and/or use of anti-arrhythmic drugs.^39^

This highlights that restoration of sinus rhythm is an important determinant of recovery of LV function if it can be achieved long term. The patients most likely to respond to this ‘rhythm control’ ablation approach are those with no evidence of myocardial abnormalities on advanced imaging and an otherwise normal ECG without significant conduction/repolarisation changes. Patients with a short history of symptoms coinciding with the onset of AF appear to have the highest chance of success using this approach, although large randomised controlled trials are required to address this formally with prolonged follow-up, for example, Catheter Ablation vs Anti-arrhythmic Drug Therapy for Atrial Fibrillation (CABANA) and Early Treatment of Atrial Fibrillation for Stroke Prevention (EAST) trials. Once advanced structural remodelling in the atria has occurred (significant LA dilation), the chances of long-term ablation success decline.

Atrial flutter is another common cause of TCMP. One study found that there was LV dysfunction in 25% of patients presenting in atrial flutter, and 57% of cases improved after ablation.^16^ Incessant atrial tachycardia is a relatively uncommon arrhythmia, but is a well-recognised cause of TCMP. It is usually caused by an automatic focus.^40–43^ Treatment of the atrial tachycardia by surgery (historical reports^26–28^ or catheter ablation^44 45^ will normalise LV function. Although the ChadsVasc score is established for anticoagulation decision making in AF and atrial flutter, given the fact that focal AT in TCMP can degenerate into AF or may promote thrombosis in the dilated ventricle/atrial appendage, some consideration should be given to anticoagulation in this context, although formal evidence is lacking at present.

Re-entrant SVTs including atrioventricular nodal reentrant tachycardia (AVNRT) and  atrioventricular reentrant tachycardia (AVRT) are usually paroxysmal and are therefore a rare cause of TCMP.^46–49^ One well-known example, however, is persistent junctional reciprocating tachycardia, which is a form of incessant AVRT, most common in paediatric cases. This usually involves a slowly conducting septal accessory pathway, but the pathway may occasionally be found elsewhere on either annulus. Ectopic atrial tachycardias are the the most common cause of TCMP in children.

A non-re-entrant form of AV nodal tachycardia may occasionally cause TCMP when a single sinus beat leads to two ventricular depolarisations with simultaneous antegrade conduction through fast and slow AV nodal pathways.^50^ If frequent enough this may lead to an incessant 1:2 tachycardia. In a review of 44 such cases described between 1970 and 2010, 8 had reduced LV function as a result.^51^ In all cases of SVT-mediated TCMP, treatment of the arrhythmia with drugs^52^ surgery^45 46^ or catheter ablation^47–49^ will reverse the LV dysfunction

## Ventricular arrhythmias

Ventricular arrhythmias causing TCMP are generally idiopathic in nature, as otherwise they may be classified as being part of the disease process leading to both arrhythmias and LV dysfunction. Thus, sustained monomorphic VT rarely causes TCMP, as it is more often associated with pre-existing structural heart disease. Idiopathic VT, if persistent or sufficiently frequent, may result in reversible LV dysfunction.^53–55^ It most often arises from the right ventricular outflow tract. One study has shown that 11% of patients presenting with frequent PVCs also had sustained monomorphic VT, and 7% of these had TCMP. 56 LV dysfunction will usually normalise following ablation of the ectopic focus.^54 55 57^

## Effects of PVCs on LV function and efficacy of CRT

**Figure 5:**
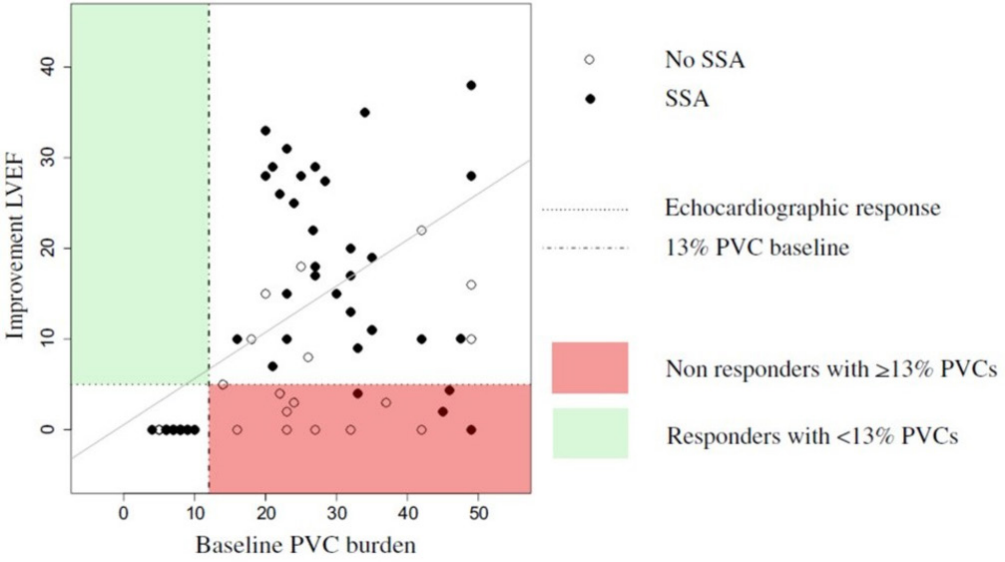
Premature ventricular complex (PVC) burden threshold likely to identify cases most likely to ablation to improve left ventricular function. A 13% baseline PVC burden had 100% sensitivity and 85% specificity to predict an absolute increase ≥5% in LV ejection fraction (LVEF) after sustained successful ablation (SSA). From Penela *et al*.^61^

Another area of much investigation and debate is the role of PVCs in promoting LV dysfunction. This issue has been addressed by several studies examining PVC burden and the role of ablation with LV impairment ranging from PVC frequency thresholds of 5–30 000 beats in 24 hours.^13 14^ A high PVC burden has been variably defined as ranging from >10 000 to 25 000 PVCs/day and as >10% to 24% of total heartbeats/day.^58–60^ There appears to be a threshold burden of ~10 000 PVCs/day for developing TCMP. A recent paper identified a threshold of 13%, which equates to a burden of approximately 30 000/24 hour (figure 5). Interestingly, ablation of >13% baseline PVC burden had 100% sensitivity and 85% specificity to predict an absolute increase in LVEF independent of evidence of structural heart disease on imaging.^61^ A number of studies have examined factors most likely to result in LV dysfunction including lack of symptoms, male sex, increased body mass index, higher PVC coupling interval dispersion, interpolated PVCs and presence of retrograde P waves. A QRS width >150 ms or epicardial origin appears a significant determinant probably because of the greater degree of dyssynchrony it promotes^62 63^ (see figure 3B—right ventricular inflow tract PVCs).

The question of whether to ablate asymptomatic monomorphic PVCs when the burden is high should be discussed carefully with the patient as PVC ablation particularly in the right ventricular outflow tract does carry a small but important risk of tamponade (<1%) and even death. Since PVC ablation restores normal LV function in the structurally normal heart, a pragmatic approach in these cases is simply to monitor the patient for signs of LV impairment and only consider ablation should this be the case. This is because isolated PVCs have an excellent prognosis in the normal hearts with preserved function anyway, so there is no reason to intervene unless significant dysfunction develops. Recently, Bhushan and Asirvatham have proposed criteria regarding PVC-induced cardiomyopathy. They suggest that otherwise young healthy individuals, without abnormal cardiovascular substrate having over 20 000 PVCs per day, no more than two PVC morphologies, PVCs originating from outflow tracts or from the fascicles and with preserved myocardial wall thickness are the best candidates for presumption of a PVC-induced cardiomyopathy diagnosis.^5^ The most prominent predictor of cardiomyopathy in patients with frequent PVCs appears to be the daily burden of PVCs. Ventricular function can improve if the PVC burden is reduced to <5000/day.^64^ This is an important target when elimination of all PVCs may not be possible, especially in the context of multifocal PVCs; overall, the efficacy of ablation is 70%–90%.

**Figure 6:**
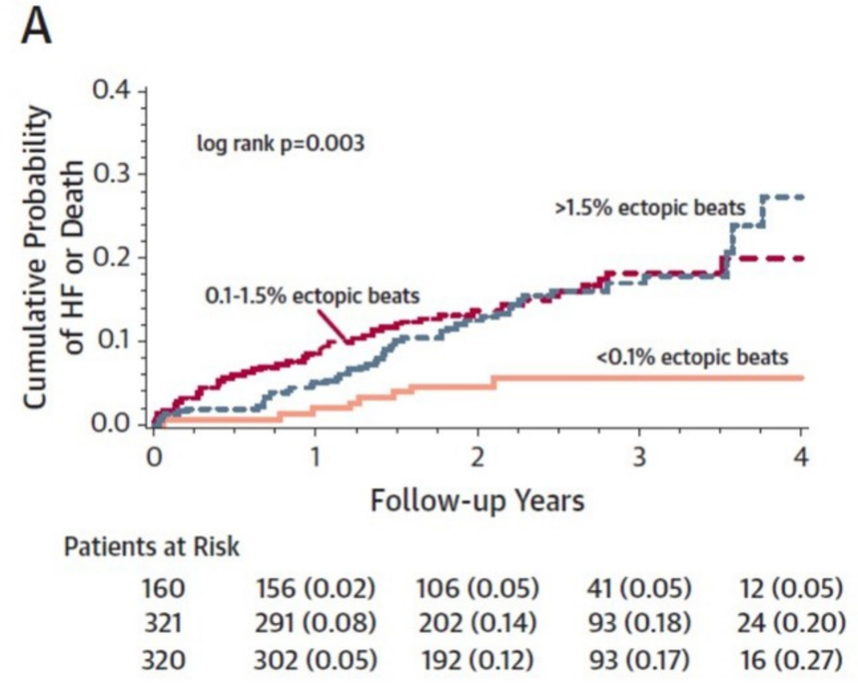
Premature ventricular complex burden effects on cardiac resynchronisation response. From Ruwald *et al*.^65^

The role of PVCs in promoting LV impairment is becoming evident in determining the response to CRT.^65^ Burdens as low as >0.1% result in significant reductions in response to CRT and clinical outcomes.^65 66^ In the Multicenter Automatic Defibrillator Implantation Trial With Cardiac Resynchronization Therapy trial, patients with as little as >0.1% ectopic beats had a lower probability of >97% CRT pacing and significantly less reverse remodelling (per cent reduction in LVESV 31±15%) than patients with <0.1% ectopic beats (per cent reduction in LVESV 39±14%; p<0.001).^65^ The risk of HF/death and ventricular arrhythmias was increased significantly in those with 0.1%–1.5% ectopic beats (HR: 3.13 and 1.84, respectively) and for >1.5% ectopic beats (HR: 2.38 and 2.74, respectively) (figure 6). This raises the question as to whether an intervention to prevent the ectopy is beneficial. A recent trial examining the effect of PVC ablation on outcomes identified a threshold of 22% ectopy likely to improve LV function with successful ablation in this context.^67^ It is important to emphasise that this strategy is best considered when there is a predominant (ideally single) PVC morphology.

**Figure 7:**
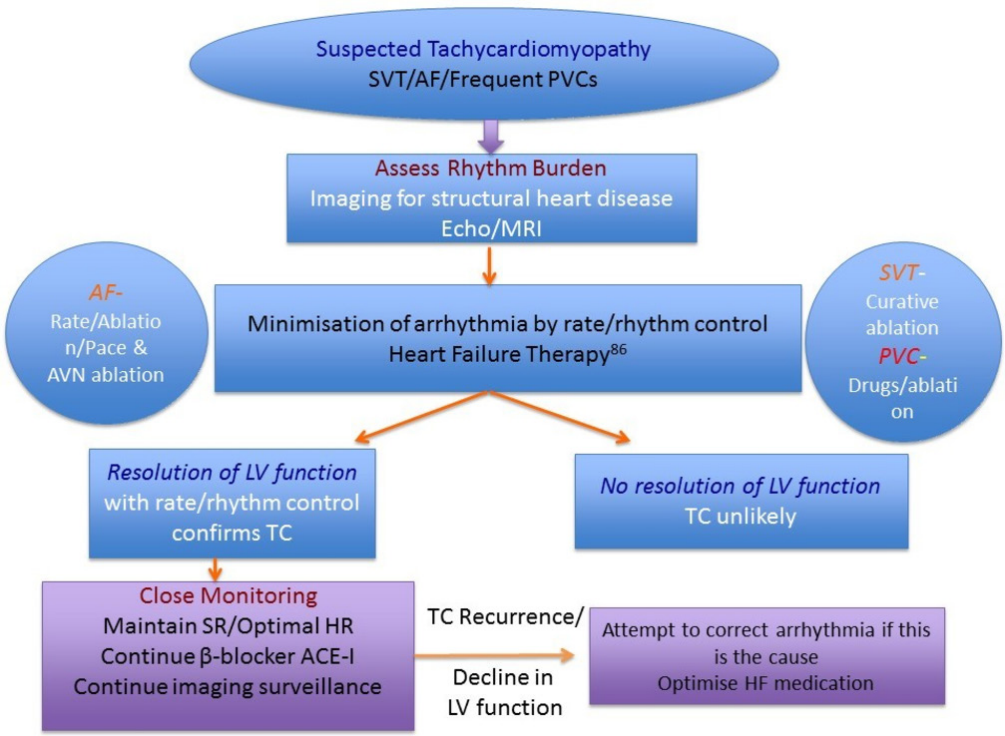
Outline of management strategy for tachycardiomyopathy. Heart failure (HF) and prevention of sudden cardiac death in structural heart disease management should be followed as per the European Guidelines. (Adapted from Gopinathannair *et al* 4; see European Guidelines on HF and prevention of sudden cardiac death (SCD)^84^). AF, atrial fibrillation; LV, left ventricular; PVC, premature ventricular complex; SVT, supraventricular tachycardia; TC, tachycardia.

## Time course 

The time course of TCMP has mostly been studied in animal models with rapid pacing. Haemodynamic abnormalities may be seen 24 hours after the onset of rapid pacing. After 1 week, there will be increased filling pressures, increased pulmonary artery pressures and decreased systemic arterial pressures. Up to 3–5 weeks, the cardiac output, EF, cardiac volume continue to worsen.^68–71^ Time to recovery from TCMP is thought to be rate and duration dependent. Often patients do not seek medical attention until they have had symptoms of heart failure for some time. In animal models, if rapid pacing is stopped, changes in filling pressures, cardiac output and systemic vascular resistance are usually reversible. EF improvement may take several weeks to normalise^72 73^ and contractile dysfunction can be seen up to 4 weeks later.^74^ End-systolic and end-diastolic volumes may still be elevated 12 weeks after cessation of pacing.^69 71 73 75^ Occasionally, EF may not normalise and some abnormalities in contractile function may persist.^69 73 76^

Similarly in humans, there is usually rapid clinical improvement. By 3 months after successful treatment of tachycardia whether by rhythm or rate control, nearly complete recovery of symptoms and LV contractility is generally observed.^10 77^ However, some more recent studies have not confirmed such significant improvements in New York Heart Association class or objective measures of cardiac function.^33 78^ In others, despite normalisation of EF, persistent LV remodelling has been demonstrated with elevated LV dimensions and volumes.^79^ This would suggest that HF therapy should be continued even once EF has recovered. Furthermore, LV hypertrophy may also develop after cessation of pacing in animal models through a postpacing response of myocytes to hypertrophic triggers, although the exact mechanisms are not well understood^5 74 80^ and this has not been seen reproducibly in humans.

## Recurrence

There is evidence to suggest that recurrent tachycardia in patients who have previously had TCMP, may result in a faster and more severe onset of TCMP than the initial presentation. In one study of 24 patients with TCMP, 5 had recurrent tachycardia associated with a rapid drop in EF and symptoms of clinical HF occurring within 6 months^81^; other small case series have reported similar findings.^82 83^ This suggests that there must be some structural cardiac abnormalities that persist after an apparent recovery in function. Therefore, maintenance of an HF treatment regimen after normalisation of EF, and continued monitoring of patients for recurrence of arrhythmia is a prudent strategy.^84^

## Sudden death

There is very little information available regarding the risk of sudden death in TCMP. HF, whatever the aetiology, leads to an arrhythmogenic substrate with repolarisation abnormalities implicated in leading to ventricular arrhythmias. Most studies have employed animal models, which have given conflicting results. A porcine model shows prolonged repolarisation, but with no deaths^85^; a canine model with repolarisation abnormalities demonstrated polymorphic VT as clinical HF worsened.^86^

A contentious issue is whether patients with resolved TCMP and normal LV function continue to have increased risk of lethal arrhythmia due to pathological remodelling. Evidence is scarce, with one case report of three patients with AF-related TCMP where EF had normalised with treatment. They all died suddenly months to years later, with normal EF and no symptoms of HF or recurrent tachycardia.^81^ One further patient with atrial flutter and TCMP who recovered with rate control, died suddenly 4 years later with no preceding symptoms.^82^

## Future directions

Although the aetiology of TCMP is well defined, there remain a number of unanswered questions in terms of identifying patients most at risk and the timing of any intervention. Furthermore, there may be specific genetic, molecular and structural features which identify patients at greatest risk that require further investigation. Also a proportion of patients do not necessarily fully respond to rhythm control or remain at risk of lethal arrhythmia. Identification of specific biomarkers to identify these individuals should be pursued through genetic, molecular profiling and imaging strategies.

## Conclusion

There is a wide range of causative arrhythmias, clinical presentations and natural histories for TCMP. The diagnosis of TCMP is difficult and a high index of suspicion is required. Given the potential for recovery with appropriate treatment, a proactive approach is recommended, whether that be rate or rhythm control. With advances in cardiac imaging and ablation techniques, it may be possible to identify patients with reversible causes of cardiomyopathy early in the disease process with those most likely to respond to rhythm control. In the meantime, a strategy attempting to minimise arrhythmia burden to assess the response even if short term, for example, DC cardioversion for AF with anti-arrhythmic therapy gives a pragmatic approach to identify such cases.

**Key messages**

- Always consider an arrhythmic aetiology as a factor contributing to cardiac dysfunction, especially in the absence of overt ventricular structural abnormalities.
- Seriously consider an early rhythm control strategy and the possibility of ablation in atrial fibrillation cases with tachycardiomyopathy.
- Carefully review ventricular ectopic burden and morphology in cardiomyopathy cases with a view to electrophysiological assessment for ablation.
- Consider ablation of monomorphic ectopics in cardiac resynchronisation non-responders if receiving <97% biventricular pacing due to ventricular ectopy.

**CME credits for Education in Heart**

Education in Heart articles are accredited for CME by various providers. To answer the accompanying multiple choice questions (MCQs) and obtain your credits, click on the 'Take the Test' link on the online version of the article. The MCQs are hosted on BMJ Learning. All users must complete a one-time registration on BMJ Learning and subsequently log in on every visit using their username and password to access modules and their CME record. Accreditation is only valid for 2 years from the date of publication. Printable CME certificates are available to users that achieve the minimum pass mark.

## References

[1] Gallagher JJ Tachycardia and cardiomyopathy: the chicken-egg dilemma revisited. J Am Coll Cardiol 1985;6:1172–3. 10.1016/S0735-1097(85)80328-4 3876364

[2] Fenelon G, Wijns W, Andries E, et al Tachycardiomyopathy: mechanisms and clinical implications. Pacing Clin Electrophysiol 1996;19:95–106. 10.1111/j.1540-8159.1996.tb04796.x 8848384

[3] Simantirakis EN, Koutalas EP, Vardas PE, et al Arrhythmia-induced cardiomyopathies: the riddle of the chicken and the egg still unanswered? Europace 2012;14:466–73. 10.1093/europace/eur348 22084300

[4] Gopinathannair R, Etheridge SP, Marchlinski FE, et al Arrhythmia-Induced cardiomyopathies: mechanisms, recognition, and Management. J Am Coll Cardiol 2015;66:1714–28. 10.1016/j.jacc.2015.08.038 26449143 PMC4733572

[5] Shinbane JS, Wood MA, Jensen DN, et al Tachycardia-induced cardiomyopathy: a review of animal models and clinical studies. J Am Coll Cardiol 1997;29:709–15. 10.1016/S0735-1097(96)00592-X 9091514

[6] Bhushan M, Asirvatham SJ The conundrum of ventricular arrhythmia and cardiomyopathy: which abnormality came first? Curr Heart Fail Rep 2009;6:7–13. 10.1007/s11897-009-0003-y 19265587

[7] Spragg DD, Akar FG, Helm RH, et al Abnormal conduction and repolarization in late-activated myocardium of dyssynchronously contracting hearts. Cardiovasc Res 2005;67:77–86. 10.1016/j.cardiores.2005.03.008 15885674

[8] Gillebert TC, Brooks N, Fontes-Carvalho R, et al ESC core curriculum for the general cardiologist (2013). Eur Heart J 2013;34:2381–411. 10.1093/eurheartj/eht234 23847131

[9] Khasnis A, Jongnarangsin K, Abela G, et al Tachycardia-induced cardiomyopathy: a review of literature. Pacing Clin Electrophysiol 2005;28:710–21. 10.1111/j.1540-8159.2005.00143.x 16008809

[10] Jeong YH, Choi KJ, Song JM, et al Diagnostic approach and treatment strategy in tachycardia-induced cardiomyopathy. Clin Cardiol 2008;31:172–8. 10.1002/clc.20161 18404727 PMC6653168

[11] Hasdemir C, Yuksel A, Camli D, et al Late gadolinium enhancement CMR in patients with tachycardia-induced cardiomyopathy caused by idiopathic ventricular arrhythmias. Pacing Clin Electrophysiol 2012;35:465–70. 10.1111/j.1540-8159.2011.03324.x 22303908

[12] Campos B, Jauregui ME, Park KM, et al New unipolar electrogram criteria to identify irreversibility of nonischemic left ventricular cardiomyopathy. J Am Coll Cardiol 2012;60:2194–204. 10.1016/j.jacc.2012.08.977 23103045

[13] Nia AM, Gassanov N, Dahlem KM, et al Diagnostic accuracy of NT-proBNP ratio (BNP-R) for early diagnosis of tachycardia-mediated cardiomyopathy: a pilot study. Clin Res Cardiol 2011;100:887–96. 10.1007/s00392-011-0319-y 21538234

[14] Allen HW Auricular fibrillation. Cal State J Med 1913;11:435–40. 18736122 PMC1640020

[15] Fujino T, Yamashita T, Suzuki S, et al Characteristics of congestive heart failure accompanied by atrial fibrillation with special reference to tachycardia-induced cardiomyopathy. Circ J 2007;71:936–40. 10.1253/circj.71.936 17526993

[16] Pizzale S, Lemery R, Green MS, et al Frequency and predictors of tachycardia-induced cardiomyopathy in patients with persistent atrial flutter. Can J Cardiol 2009;25:469–72. 10.1016/S0828-282X(09)70119-9 19668781 PMC2732374

[17] Furushima H, Chinushi M, Sugiura H, et al Radiofrequency catheter ablation for incessant atrioventricular nodal reentrant tachycardia normalized H-V block associated with tachycardia-induced cardiomyopathy. J Electrocardiol 2004;37:315–9. 10.1016/j.jelectrocard.2004.07.009 15484161

[18] Medi C, Kalman JM, Haqqani H, et al Tachycardia-mediated cardiomyopathy secondary to focal atrial tachycardia: long-term outcome after catheter ablation. J Am Coll Cardiol 2009;53:1791–7. 10.1016/j.jacc.2009.02.014 19422986

[19] Bensler JM, Frank CM, Razavi M, et al Tachycardia-mediated cardiomyopathy and the permanent form of junctional reciprocating tachycardia. Tex Heart Inst J 2010;37:695–8. 21224950 PMC3014141

[20] Krapp M, Gembruch U, Baumann P, et al Venous blood flow pattern suggesting tachycardia-induced 'cardiomyopathy' in the fetus. Ultrasound Obstet Gynecol 1997;10:32–40. 10.1046/j.1469-0705.1997.10010032.x 9263421

[21] Dhala A, Thomas JP Images in cardiovascular medicine. reversible tachycardia-induced cardiomyopathy. Circulation 1997;95:2327–8. 9142013 10.1161/01.cir.95.9.2327

[22] Sanchez C, Benito F, Moreno F, et al Reversibility of tachycardia-induced cardiomyopathy after radiofrequency ablation of incessant supraventricular tachycardia in infants. Br Heart J 1995;74:332–3. 10.1136/hrt.74.3.332 7547032 PMC484029

[23] De Giovanni JV, Dindar A, Griffith MJ, et al Recovery pattern of left ventricular dysfunction following radiofrequency ablation of incessant supraventricular tachycardia in infants and children. Heart 1998;79:588–92. 10.1136/hrt.79.6.588 10078086 PMC1728732

[24] Ju W, Yang B, Li M, et al Tachycardiomyopathy complicated by focal atrial tachycardia: incidence, risk factors, and long-term outcome. J Cardiovasc Electrophysiol 2014;25:953–7. 10.1111/jce.12428 24716793

[25] Kang KT, Etheridge SP, Kantoch MJ, et al Current management of focal atrial tachycardia in children: a multicenter experience. Circ Arrhythm Electrophysiol 2014;7:664–70. 10.1161/CIRCEP.113.001423 25015944

[26] Yokokawa M, Good E, Crawford T, et al Recovery from left ventricular dysfunction after ablation of frequent premature ventricular complexes. Heart Rhythm 2013;10:172–5. 10.1016/j.hrthm.2012.10.011 23099051

[27] Kawamura M, Badhwar N, Vedantham V, et al Coupling interval dispersion and body mass index are independent predictors of idiopathic premature ventricular complex-induced cardiomyopathy. J Cardiovasc Electrophysiol 2014;25:756–62. 10.1111/jce.12391 24612052

[28] Whipple GH, Sheffield LT, Woodman EG, et al Theophilis C, Friedman S. reversible congestive heart failure due to chronic rapid stimulation of the normal heart. Proc N Engl Cardiovasc Soc 1962.

[29] Twidale N, Sutton K, Bartlett L, et al Effects on cardiac performance of atrioventricular node catheter ablation using radiofrequency current for drug-refractory atrial arrhythmias. Pacing Clin Electrophysiol 1993;16:1275–84. 10.1111/j.1540-8159.1993.tb01714.x 7686657

[30] Heinz G, Siostrzonek P, Kreiner G, et al Improvement in left ventricular systolic function after successful radiofrequency his bundle ablation for drug refractory, chronic atrial fibrillation and recurrent atrial flutter. Am J Cardiol 1992;69:489–92. 10.1016/0002-9149(92)90991-7 1736612

[31] Manolis AG, Katsivas AG, Lazaris EE, et al Ventricular performance and quality of life in patients who underwent radiofrequency AV junction ablation and permanent pacemaker implantation due to medically refractory atrial tachyarrhythmias. J Interv Card Electrophysiol 1998;2:71–6. 10.1023/A:1009721008761 9869999

[32] Redfield MM, Kay GN, Jenkins LS, et al Tachycardia-related cardiomyopathy: a common cause of ventricular dysfunction in patients with atrial fibrillation referred for atrioventricular ablation. Mayo Clin Proc 2000;75:790–5. 10.4065/75.8.790 10943231

[33] Brignole M, Gianfranchi L, Menozzi C, et al Influence of atrioventricular junction radiofrequency ablation in patients with chronic atrial fibrillation and flutter on quality of life and cardiac performance. Am J Cardiol 1994;74:242–6. 10.1016/0002-9149(94)90364-6 8037128

[34] Edner M, Caidahl K, Bergfeldt L, et al Prospective study of left ventricular function after radiofrequency ablation of atrioventricular junction in patients with atrial fibrillation. Br Heart J 1995;74:261–7. 10.1136/hrt.74.3.261 7547020 PMC484016

[35] Natale A, Zimerman L, Tomassoni G, et al Impact on ventricular function and quality of life of transcatheter ablation of the atrioventricular junction in chronic atrial fibrillation with a normal ventricular response. Am J Cardiol 1996;78:1431–3. 10.1016/S0002-9149(97)89296-X 8970421

[36] Hunter RJ, Berriman TJ, Diab I, et al A randomized controlled trial of catheter ablation versus medical treatment of atrial fibrillation in heart failure (the CAMTAF trial). Circ Arrhythm Electrophysiol 2014;7:31–8. 10.1161/CIRCEP.113.000806 24382410

[37] Khan MN, Jaïs P, Cummings J, et al Pulmonary-vein isolation for atrial fibrillation in patients with heart failure. N Engl J Med 2008;359:1778–85. 10.1056/NEJMoa0708234 18946063

[38] Di Biase L, Mohanty P, Mohanty S, et al Ablation Versus Amiodarone for treatment of Persistent Atrial Fibrillation in Patients with Congestive Heart failure and an implanted device: results from the AATAC Multicenter Randomized Trial. Circulation 2016;133:1637–44. 10.1161/CIRCULATIONAHA.115.019406 27029350

[39] Ganesan AN, Nandal S, Lüker J, et al Catheter ablation of atrial fibrillation in patients with concomitant left ventricular impairment: a systematic review of efficacy and effect on ejection fraction. Heart Lung Circ 2015;24:270–80. 10.1016/j.hlc.2014.09.012 25456506

[40] Scheinman MM, Basu D, Hollenberg M, et al Electrophysiologic studies in patients with persistent atrial tachycardia. Circulation 1974;50:266–73. 10.1161/01.CIR.50.2.266 4846635

[41] Bertil Olsson S, Blomström P, Sabel KG, et al Incessant ectopic atrial tachycardia: successful surgical treatment with regression of dilated cardiomyopathy picture. Am J Cardiol 1984;53:1465–6. 10.1016/S0002-9149(84)91329-8 6720596

[42] Gillette PC, Smith RT, Garson A, et al Chronic supraventricular tachycardia. A curable cause of congestive cardiomyopathy. JAMA 1985;253:391–2. 3965793 10.1001/jama.253.3.391

[43] Gillette PC, Wampler DG, Garson A, et al Treatment of atrial automatic tachycardia by ablation procedures. J Am Coll Cardiol 1985;6:405–9. 10.1016/S0735-1097(85)80179-0 4019927

[44] Cruz FE, Cheriex EC, Smeets JL, et al Reversibility of tachycardia-induced cardiomyopathy after cure of incessant supraventricular tachycardia. J Am Coll Cardiol 1990;16:739–44. 10.1016/0735-1097(90)90368-Y 2387945

[45] Chiladakis JA, Vassilikos VP, Maounis TN, et al Successful radiofrequency catheter ablation of automatic atrial tachycardia with regression of the cardiomyopathy picture. Pacing Clin Electrophysiol 1997;20:953–9. 10.1111/j.1540-8159.1997.tb05499.x 9127401

[46] Packer DL, Bardy GH, Worley SJ, et al Tachycardia-induced cardiomyopathy: a reversible form of left ventricular dysfunction. Am J Cardiol 1986;57:563–70. 10.1016/0002-9149(86)90836-2 3953440

[47] Fishberger SB, Colan SD, Saul JP, et al Myocardial mechanics before and after ablation of chronic tachycardia. Pacing Clin Electrophysiol 1996;19:42–9. 10.1111/j.1540-8159.1996.tb04789.x 8848376

[48] Aguinaga L, Primo J, Anguera I, et al Long-term follow-up in patients with the permanent form of junctional reciprocating tachycardia treated with radiofrequency ablation. Pacing Clin Electrophysiol 1998;21:2073–8. 10.1111/j.1540-8159.1998.tb01126.x 9826859

[49] Corey WA, Markel ML, Hoit BD, et al Regression of a dilated cardiomyopathy after radiofrequency ablation of incessant supraventricular tachycardia. Am Heart J 1993;126:1469–73. 10.1016/0002-8703(93)90549-O 8249807

[50] Wu D, Denes P, Dhingra R, et al New manifestations of dual A-V nodal pathways. Eur J Cardiol 1975;2:459–66. 1126354

[51] Wang NC Dual atrioventricular nodal nonreentrant tachycardia: a systematic review. Pacing Clin Electrophysiol 2011;34:1671–81. 10.1111/j.1540-8159.2011.03218.x 21950798

[52] Leman RB, Gillette PC, Zinner AJ, et al Resolution of congestive cardiomyopathy caused by supraventricular tachycardia using amiodarone. Am Heart J 1986;112:622–4. 10.1016/0002-8703(86)90535-1 3751876

[53] Anselme F, Boyle N, Josephson M, et al Incessant fascicular tachycardia: a cause of arrhythmia induced cardiomyopathy. Pacing Clin Electrophysiol 1998;21:760–3. 10.1111/j.1540-8159.1998.tb00135.x 9584309

[54] Vijgen J, Hill P, Biblo LA, et al Tachycardia-induced cardiomyopathy secondary to right ventricular outflow tract ventricular tachycardia: improvement of left ventricular systolic function after radiofrequency catheter ablation of the arrhythmia. J Cardiovasc Electrophysiol 1997;8:445–50. 10.1111/j.1540-8167.1997.tb00811.x 9106431

[55] Singh B, Kaul U, Talwar KK, et al Reversibility of **“**tachycardia induced cardiomyopathy” following the cure of idiopathic left ventricular tachycardia using radiofrequency energy. Pacing Clin Electrophysiol 1996;19:1391–2. 10.1111/j.1540-8159.1996.tb04222.x 8880807

[56] Hasdemir C, Ulucan C, Yavuzgil O, et al Tachycardia-induced cardiomyopathy in patients with idiopathic ventricular arrhythmias: the incidence, clinical and electrophysiologic characteristics, and the predictors. J Cardiovasc Electrophysiol 2011;22:663–8. 10.1111/j.1540-8167.2010.01986.x 21235667

[57] Grimm W, Menz V, Hoffmann J, et al Reversal of tachycardia induced cardiomyopathy following ablation of repetitive monomorphic right ventricular outflow tract tachycardia. Pacing Clin Electrophysiol 2001;24:166–71. 10.1046/j.1460-9592.2001.00166.x 11270695

[58] Baman TS, Lange DC, Ilg KJ, et al Relationship between burden of premature ventricular complexes and left ventricular function. Heart Rhythm 2010;7:865–9. 10.1016/j.hrthm.2010.03.036 20348027

[59] Kanei Y, Friedman M, Ogawa N, et al Frequent premature ventricular complexes originating from the right ventricular outflow tract are associated with left ventricular dysfunction. Ann Noninvasive Electrocardiol 2008;13:81–5. 10.1111/j.1542-474X.2007.00204.x 18234010 PMC6932157

[60] Niwano S, Wakisaka Y, Niwano H, et al Prognostic significance of frequent premature ventricular contractions originating from the ventricular outflow tract in patients with normal left ventricular function. Heart 2009;95:1230–7. 10.1136/hrt.2008.159558 19429571

[61] Penela D, Van Huls Van Taxis C, Aguinaga L, et al Neurohormonal, structural, and functional recovery pattern after premature ventricular complex ablation is independent of structural heart disease status in patients with depressed left ventricular ejection fraction: a prospective multicenter study. J Am Coll Cardiol 2013;62:1195–202. 10.1016/j.jacc.2013.06.012 23850913

[62] Carballeira Pol L, Deyell MW, Frankel DS, et al Ventricular premature depolarization QRS duration as a new marker of risk for the development of ventricular premature depolarization-induced cardiomyopathy. Heart Rhythm 2014;11:299–306. 10.1016/j.hrthm.2013.10.055 24184787

[63] Sadron Blaye-Felice M, Hamon D, Sacher F, et al Premature ventricular contraction-induced cardiomyopathy: related clinical and electrophysiologic parameters. Heart Rhythm 2016;13:103–10. 10.1016/j.hrthm.2015.08.025 26296327

[64] Mountantonakis SE, Frankel DS, Gerstenfeld EP, et al Reversal of outflow tract ventricular premature depolarization-induced cardiomyopathy with ablation: effect of residual arrhythmia burden and preexisting cardiomyopathy on outcome. Heart Rhythm 2011;8:1608–14. 10.1016/j.hrthm.2011.04.026 21699837

[65] Ruwald MH, Mittal S, Ruwald AC, et al Association between frequency of atrial and ventricular ectopic beats and biventricular pacing percentage and outcomes in patients with cardiac resynchronization therapy. J Am Coll Cardiol 2014;64:971–81. 10.1016/j.jacc.2014.06.1177 25190230

[66] Hayes DL, Boehmer JP, Day JD, et al Cardiac resynchronization therapy and the relationship of percent biventricular pacing to symptoms and survival. Heart Rhythm 2011;8:1469–75. 10.1016/j.hrthm.2011.04.015 21699828

[67] Lakkireddy D, Di Biase L, Ryschon K, et al Radiofrequency ablation of premature ventricular ectopy improves the efficacy of cardiac resynchronization therapy in nonresponders. J Am Coll Cardiol 2012;60:1531–9. 10.1016/j.jacc.2012.06.035 22999718

[68] Riegger AJG, Liebau G The Renin-Angiotensin-Aldosterone System, antidiuretic hormone and sympathetic nerve activity in an experimental Model of congestive Heart failure in the dog. Clin Sci 1982;62:465–9. 10.1042/cs0620465 7075144

[69] Damiano RJ, Tripp HF, Asano T, et al Left ventricular dysfunction and dilatation resulting from chronic supraventricular tachycardia. J Thorac Cardiovasc Surg 1987;94:135–43. 3599999

[70] Ohno M, Cheng CP, Little WC, et al Mechanism of altered patterns of left ventricular filling during the development of congestive heart failure. Circulation 1994;89:2241–50. 10.1161/01.CIR.89.5.2241 8181149

[71] Howard RJ, Moe GW, Armstrong PW, et al Sequential echocardiographic-Doppler assessment of left ventricular remodelling and mitral regurgitation during evolving experimental heart failure. Cardiovasc Res 1991;25:468–74. 10.1093/cvr/25.6.468 1889061

[72] Howard RJ, Stopps TP, Moe GW, et al Recovery from heart failure: structural and functional analysis in a canine model. Can J Physiol Pharmacol 1988;66:1505–12. 10.1139/y88-246 3228785

[73] Moe GW, Stopps TP, Howard RJ, et al Early recovery from heart failure: insights into the pathogenesis of experimental chronic pacing-induced heart failure. J Lab Clin Med 1988;112:426–32. 3171352

[74] Spinale FG, Holzgrefe HH, Mukherjee R, et al LV and myocyte structure and function after early recovery from tachycardia-induced cardiomyopathy. Am J Physiol 1995;268:H836–47. 7864211 10.1152/ajpheart.1995.268.2.H836

[75] Morgan DE, Tomlinson CW, Qayumi AK, et al Evaluation of ventricular contractility indexes in the dog with left ventricular dysfunction induced by rapid atrial pacing. J Am Coll Cardiol 1989;14:489–95. 10.1016/0735-1097(89)90206-4 2754134

[76] Yamamoto K, Burnett JC, Meyer LM, et al Ventricular remodeling during development and recovery from modified tachycardia-induced cardiomyopathy model. Am J Physiol 1996;271:R1529–34. 8997349 10.1152/ajpregu.1996.271.6.R1529

[77] Kay GN, Ellenbogen KA, Giudici M, et al The Ablate and Pace Trial: a prospective study of catheter ablation of the AV conduction system and permanent pacemaker implantation for treatment of atrial fibrillation. APT investigators. J Interv Card Electrophysiol 1998;2:121–35. 10.1023/A:1009795330454 9870004

[78] Weerasooriya R, Davis M, Powell A, et al The australian intervention Randomized Control of Rate in Atrial Fibrillation Trial (AIRCRAFT). J Am Coll Cardiol 2003;41:1697–702. 10.1016/S0735-1097(03)00338-3 12767649

[79] Dandamudi G, Rampurwala AY, Mahenthiran J, et al Persistent left ventricular dilatation in tachycardia-induced cardiomyopathy patients after appropriate treatment and normalization of ejection fraction. Heart Rhythm 2008;5:1111–4. 10.1016/j.hrthm.2008.04.023 18675220

[80] Tomita M, Spinale FG, Crawford FA, et al Changes in left ventricular volume, mass, and function during the development and regression of supraventricular tachycardia-induced cardiomyopathy. disparity between recovery of systolic versus diastolic function. Circulation 1991;83:635–44. 10.1161/01.CIR.83.2.635 1991381

[81] Nerheim P, Birger-Botkin S, Piracha L, et al Heart failure and sudden death in patients with tachycardia-induced cardiomyopathy and recurrent tachycardia. Circulation 2004;110:247–52. 10.1161/01.CIR.0000135472.28234.CC 15226218

[82] Watanabe H, Okamura K, Chinushi M, et al Clinical characteristics, treatment, and outcome of tachycardia induced cardiomyopathy. Int Heart J 2008;49:39–47. 10.1536/ihj.49.39 18360063

[83] Kieny JR, Sacrez A, Facello A, et al Increase in radionuclide left ventricular ejection fraction after cardioversion of chronic atrial fibrillation in idiopathic dilated cardiomyopathy. Eur Heart J 1992;13:1290–5. 10.1093/oxfordjournals.eurheartj.a060351 1396842

[84] Ponikowski P, Voors AA, Anker SD, et al 2016 ESC guidelines for the diagnosis and treatment of acute and chronic heart failure: the Task Force for the diagnosis and treatment of acute and chronic heart failure of the european Society of Cardiology (ESC) Developed with the special contribution of the Heart failure association (HFA) of the ESC. Eur Heart J 2016 37:2129–200. 10.1093/eurheartj/ehw128 27206819

[85] Lacroix D, Gluais P, Marquié C, et al Repolarization abnormalities and their arrhythmogenic consequences in porcine tachycardia-induced cardiomyopathy. Cardiovasc Res 2002;54:42–50. 10.1016/S0008-6363(02)00236-5 12062360

[86] Pak PH, Nuss HB, Tunin RS, et al Repolarization abnormalities, arrhythmia and sudden death in canine tachycardia-induced cardiomyopathy. J Am Coll Cardiol 1997;30:576–84. 10.1016/S0735-1097(97)00193-9 9247535

